# Silent Suffering: Clinical and Electrophysiological Profiling of Peripheral Neuropathy in Hemodialysis Patients in Nepal

**DOI:** 10.7759/cureus.92398

**Published:** 2025-09-15

**Authors:** Rayana Shrestha, Mukunda P Kafle, Nava R Sharma, Abhishesh Wagle, Madalasa Pokhrel, Dibya Singh Shah

**Affiliations:** 1 Department of Internal Medicine, Tribhuvan University Teaching Hospital (TUTH), Kathmandu, NPL; 2 Department of Nephrology and Transplantation Medicine, Tribhuvan University Teaching Hospital (TUTH), Kathmandu, NPL; 3 Internal Medicine, Maimonides Medical Center, Brooklyn, USA; 4 Medicine, Manipal College of Medical Science, Pokhara, NPL; 5 Infectious Disease, Maimonides Medical Center, Brooklyn, USA

**Keywords:** acquired peripheral neuropathy, ckd complications, diabetic neuropathy, end stage renal disease (esrd), uremic neuropathy

## Abstract

Background and objective

Peripheral neuropathy (PN) is a common and debilitating complication of chronic kidney disease (CKD), particularly in patients undergoing hemodialysis (CKD stage 5 on dialysis, CKD5D). This study aimed to determine the prevalence of PN among CKD5D patients at a tertiary care center in Nepal and to characterize its clinical and electrophysiological features.

Methodology

A single-center, observational, cross-sectional study was conducted at Tribhuvan University Teaching Hospital (TUTH) from November 2020 to October 2021. Adult patients (≥18 years) with CKD5D undergoing maintenance hemodialysis were included. Patients with a prior renal transplant or known neurological disorders were excluded. Peripheral PN was assessed by using the Michigan Neuropathy Screening Instrument (MNSI) questionnaire and physical examination, and confirmed by nerve conduction studies (NCS). Data were analyzed using SPSS® Statistics version 25 (IBM Corp., Armonk, NY), and ethical approval was obtained from the Institutional Review Board.

Results

Among the 116 enrolled patients, 80 were male (68.9%), with a mean age of 44.9 ± 15.47 years. The etiology of CKD was undetermined in 76 patients (65.5%), followed by diabetic kidney disease in 32 patients (27.6%). The mean duration of hemodialysis was 14 months, and 91 patients (78.4%) received eight hours of dialysis per week. PN was detected in 106 patients (91.4%), including all 35 diabetic participants (100%) and 71 out of 81 non-diabetic patients (87.7%). Based on the MNSI questionnaire, 14 patients (12.1%) reported neuropathic symptoms, while 60 (51.7%) had signs of PN on physical examination. Distal sensorimotor axonal neuropathy was the predominant pattern observed. The most frequently affected nerves were the common peroneal nerve (motor) and the sural nerve (sensory). Notably, lower serum albumin levels were significantly associated with the presence of PN (p=0.017).

Conclusions

PN is highly prevalent in CKD5D patients in Nepal, including among non-diabetics. Routine screening using clinical tools such as the MNSI, complemented by electrodiagnostic testing, may facilitate early detection and guide management strategies.

## Introduction

Chronic kidney disease (CKD) is an increasingly prevalent global health concern, affecting approximately 9.1% (697.5 million) of the global population [1]. A nationally representative cross-sectional survey of 12,109 Nepalese adults between 2016 and 2018 using multistage cluster sampling reported an overall CKD prevalence of 6.0% (95% CI: 5.5-6.6) among individuals aged 20 years and older [2]. Among the various complications associated with CKD, peripheral neuropathy (PN) is one of the most frequent, particularly in patients receiving hemodialysis [3]. The pathogenesis of PN in CKD is multifactorial and includes the accumulation of uremic toxins, electrolyte and metabolic disturbances, vitamin deficiencies, and retention of metabolic waste products. Uremic neuropathy was first described by Kussmaul in 1863 and later elaborated by Asbury in the early 20th century [4]. Clinically, it presents as symmetrical burning sensations in the feet followed by progressive numbness and weakness, most commonly affecting the distal extremities.

Electrophysiological studies by Bolton et al. in 1970 demonstrated a correlation between the severity of renal dysfunction and reductions in nerve conduction velocity [5]. Later, Dyck and co-investigators characterized uremic neuropathy in greater detail using both nerve conduction studies and histopathological analysis [6]. PN contributes significantly to morbidity in CKD and has a substantial negative impact on quality of life. Although it most commonly manifests when the estimated glomerular filtration rate (eGFR) falls below 12 12 mL/min/1.73 m², early subclinical changes may occur before this threshold [7]. Recent investigations have highlighted the role of middle molecules, which are uremic toxins of intermediate molecular weight, in the development of PN. These toxins are often inadequately cleared by standard dialysis techniques and may account for the persistent prevalence of PN among patients undergoing long-term hemodialysis [8,9].

Despite ongoing advancements in nephrology and dialysis, effective treatment options for uremic neuropathy remain limited. Although PN is a well-recognized neurological complication in patients with end-stage renal disease, there is a lack of comprehensive research focusing on its clinical and electrophysiological characteristics in the Nepalese population. Current data from Nepal are limited, emphasizing the need for population-specific studies to better understand the burden and manifestations of PN in CKD. The primary aim of this study was to determine the prevalence of PN among patients with CKD receiving maintenance hemodialysis. The secondary objectives were to (1) compare the frequency of peripheral neuropathy between diabetic and non-diabetic hemodialysis patients; (2) identify the most common type of PN; (3) evaluate the relationship of PN with the duration and frequency of dialysis; and (4) analyze the clinical and demographic characteristics of patients with PN.

## Materials and methods

Study design and setting

This was a single-center, observational, cross-sectional study conducted at Tribhuvan University Teaching Hospital in Kathmandu, Nepal, over a period of 12 months from November 2020 to October 2021. Adult patients aged 18 years and above who had been diagnosed with CKD stage 5 on maintenance hemodialysis (CKD5D), as defined by the Kidney Disease Improving Global Outcomes (KDIGO) 2012 clinical practice guidelines, were eligible for inclusion [10]. Only those who provided informed written consent were enrolled in the study. Patients were excluded if they had a prior history of any known neurological disorder, diagnosed hypothyroidism, a history of renal transplantation, or were undergoing peritoneal dialysis at the time of evaluation.

Study protocol

**Figure 1:**
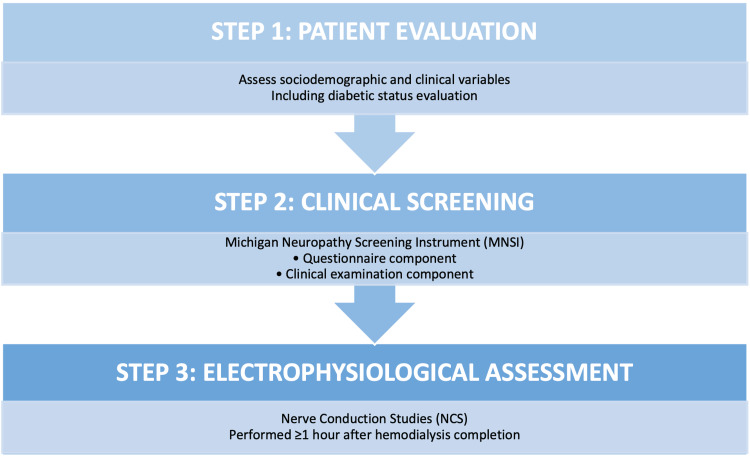
Study protocol

The study was conducted in three structured phases to systematically assess the presence and characteristics of PN in patients with CKD receiving maintenance hemodialysis, as shown in Figure 1.

All eligible participants underwent a detailed initial evaluation in which sociodemographic and clinical data were collected. This included age, sex, duration of dialysis, comorbid conditions, and other relevant medical history. The most common relevant medical histories were hypertension, diabetes mellitus, and cardiovascular disease. Special attention was given to assessing diabetic status, including duration of diabetes, glycemic control, and presence of diabetic complications. Clinical records and patient interviews were used to gather comprehensive baseline information.

Following initial evaluation, patients underwent clinical screening for peripheral neuropathy using the Michigan Neuropathy Screening Instrument (MNSI) [11]. This validated tool includes two components: a questionnaire-based assessment, consisting of patient-reported symptoms such as numbness, tingling, burning sensation, and sensitivity in the lower limbs [11]; a structured clinical examination, evaluating signs such as appearance of the feet, ulceration, ankle reflexes, and vibration perception using a tuning fork [11]. A positive MNSI score was used as a preliminary indicator for PN and guided further assessment [11].

All patients subsequently underwent electrophysiological evaluation using nerve conduction studies (NCS) [12]. These studies were performed at least one hour after the completion of hemodialysis to minimize the influence of acute dialysis-related fluid and electrolyte shifts on nerve conduction parameters [12]. Standard techniques were used to assess both motor and sensory nerve conduction velocities, amplitudes, and latencies in the upper and lower limbs. The studies were interpreted by a trained neurologist, and findings were classified according to established criteria for PN.

Michigan Neuropathy Screening Instrument (MNSI)

The MNSI is a validated tool used for the detection of distal symmetrical PN [11]. It comprises two components: Part A involves a 15-item self-administered questionnaire that assesses subjective symptoms related to neuropathy, while Part B entails a structured lower extremity examination conducted by a clinician [11]. The physical examination includes inspection of the feet, assessment of vibratory sensation using a 128 Hz tuning fork, and evaluation of ankle reflexes.

Although originally developed for the assessment of diabetic PN, the MNSI has been applied across various patient populations, including those with CKD [11]. Its dual-component design offers the advantage of combining subjective symptomatology with objective clinical findings, enhancing diagnostic sensitivity and specificity [11]. In this study, PN was deemed present if the MNSI questionnaire score was 7 or higher, or if the clinical examination score was 3 or higher.

Electrophysiological classification

PN was further classified based on findings from NCS, which were performed according to established protocols [12]. Based on electrophysiological characteristics, neuropathies are categorized into three types. Axonal neuropathy is defined by a reduction in compound muscle action potential (CMAP) amplitudes, while conduction velocities remain at or above 75% of the lower limit of normal and distal latencies are within 130% of the upper normal limit [12]. In contrast, demyelinating neuropathy is characterized by preserved CMAP amplitudes, with conduction velocities falling below 75% of the normal range and/or distal latencies exceeding 130% of the upper normal limit [12]. Mixed neuropathy is diagnosed when features of both axonal and demyelinating patterns are present on NCS [12].

Data analysis

Data were initially compiled and organized using Microsoft Excel and subsequently analyzed using SPSS® Statistics version 25 (IBM Corp., Armonk, NY) [13]. Descriptive statistics were used to summarize the demographic and clinical characteristics of the study population. Associations between PN and independent categorical variables were assessed using the chi-square test. The relationship between MNSI scores and nerve conduction study outcomes was evaluated using Spearman's correlation coefficient. A p-value of less than 0.05 was considered statistically significant.

## Results

Sociodemographic profile

The study included 116 patients with CKD undergoing dialysis. The majority were aged 40-60 years (n=49, 42.2%), followed by 20-40 years (n=43, 37%). The male-to-female ratio was 2.2:1, with 80 males (68.9%) and 36 females (31.1%). Most participants were Hindu (n=91, 78.44%) and married (n=96, 82.75%). Equal proportions of patients (n=58, 50%) were receiving erythropoiesis-stimulating agents (ESA). Regarding occupation, 38 (32.7%) were unemployed, followed by 31 (26.7%) homemakers. The vast majority (n=111, 95.7%) consumed a non-vegetarian diet. Of note, 35 (30.17%) were diabetic and 81 (69.83%) were non-diabetic. Table 1 provides a detailed breakdown of these characteristics.

**Table 1:** Sociodemographic characteristics of CKD5D patients CKD5D: chronic kidney disease stage 5 on dialysis; ESA: erythropoiesis-stimulating agent

Variable	Category	Frequency (n)	Percent (%)
Age (years)	<20	2	1.72
	20–40	43	37
	40–60	49	42.2
	>60	22	19.08
Gender	Male	80	68.9
	Female	36	31.1
Religion	Hindu	91	78.44
	Buddhist	22	18.9
	Muslim	3	2.66
Marital status	Married	96	82.75
	Unmarried	20	17.35
ESA use	Yes	58	50
	No	58	50
Occupation	Homemaker	31	26.7
	Farmer	14	12
	Driver	5	4.3
	Official/bank/business	9	7.7
	Student	7	6
	Laborer	5	4.3
	Retired officer/army	4	3.4
	Foreign migrant	3	2.5
	Unemployed	38	32.7
Diet	Non-vegetarian	111	95.7
	Vegetarian	5	4.3

Clinicoetiological profile

**Figure 2:**
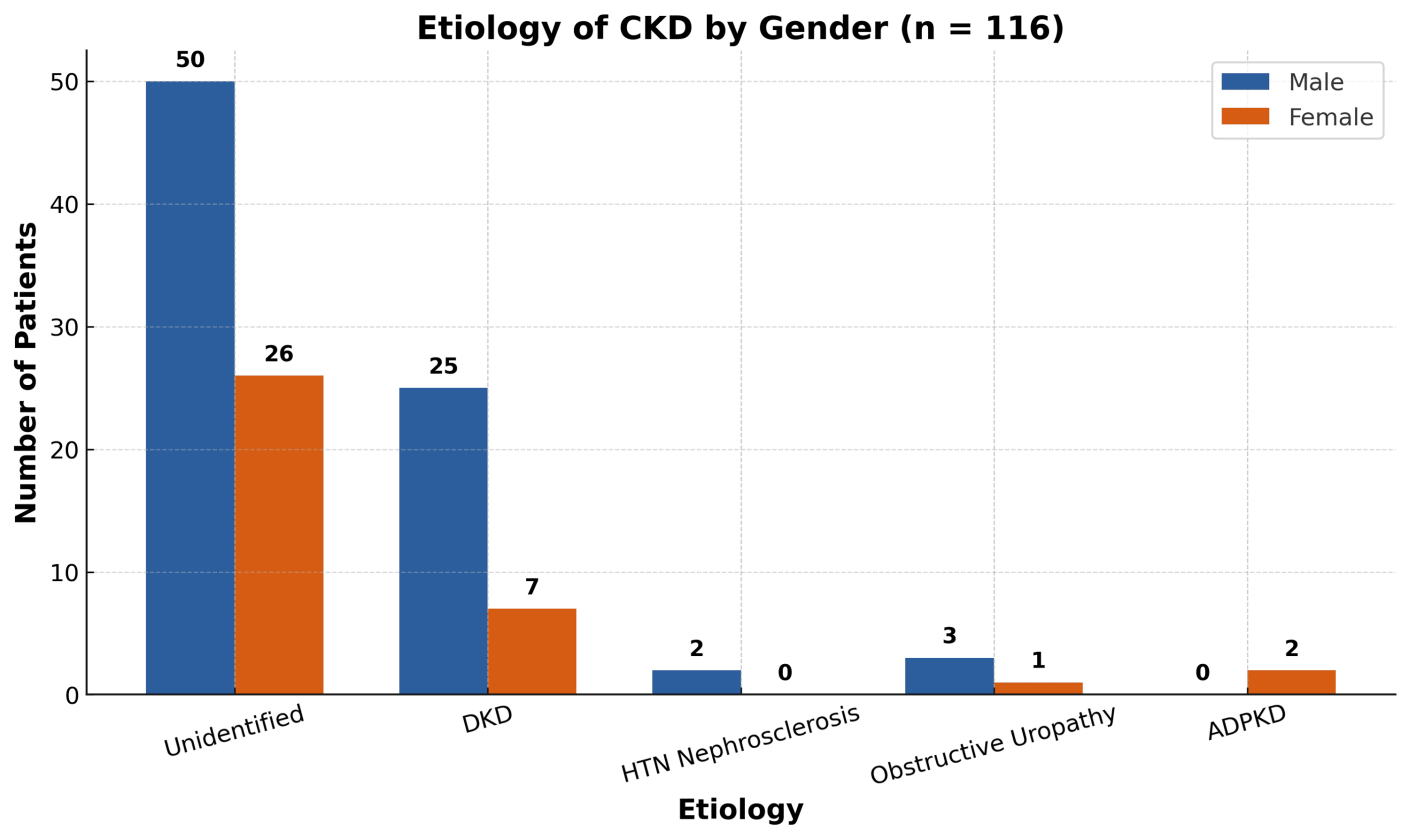
Etiology of CKD5D CKD5D: chronic kidney disease stage 5 on dialysis; DKD: diabetic kidney disease; HTN: hypertension; ADPKD: autosomal dominant polycystic kidney disease

Regarding etiology, it was unknown in the majority of patients (n=76, 65.5%). This was followed by diabetic kidney disease (DKD), which was the second leading cause, observed in 32 (27.58%) patients. The causes of CKD among the study participants are illustrated in Figure 2. Less common etiologies included hypertensive nephrosclerosis (n=2, 1.7%), obstructive uropathy (n=4, 3.4%), and autosomal dominant polycystic kidney disease (ADPKD) (n=2, 1.7%).

The laboratory investigation findings of the patients are shown in Table 2.

**Table 2:** Laboratory results of CKD5D patients CKD5D: chronic kidney disease stage 5 on dialysis; SD: standard deviation; iPTH: intact parathyroid hormone

Parameter	Mean ± SD
Hemoglobin (g/dL)	8.48 ± 1.67
Platelets (/µL)	171,596 ± 84,713
Creatinine (µmol/L)	1026 ± 449
Urea (mmol/L)	30 ± 12.64
Sodium (mEq/L)	132 ± 10.7
Potassium (mEq/L)	4.9 ± 0.88
Calcium (mmol/L)	1.86 ± 1.32
Phosphorus (mg/dL)	6.08 ± 1.97
Vitamin D (ng/mL)	24.8 ± 18
Albumin (mg/dL)	35.56 ± 5.08
Vitamin B12 (pg/mL)	512.26 ± 188.6
iPTH (pg/mL)	390 ± 277

Prevalence of peripheral neuropathy

Nerve conduction studies revealed that 91.4% (n=106) of patients had evidence of PN (Table 3). Among these, 15.5% (n=18) had involvement of a single nerve, while 75.9% (n=88) had multiple nerves affected.

**Table 3:** Frequency of peripheral neuropathy by number of nerves involved

Number of nerves involved	Frequency (n)	Percent (%)
No nerves involved	10	8.60
1 nerve involved	18	15.50
>1 nerve involved	88	75.90
Total	116	100

Pattern of neuropathy

Axonal neuropathy was the most frequent pattern observed in both the upper and lower limbs among patients. The detailed distribution of neuropathy types is summarized in Table 4.

**Table 4:** Patterns of neuropathy in CKD5D patients CKD5D: chronic kidney disease stage 5 on dialysis

Pattern of neuropathy	Upper limb motor	Upper limb sensory	Lower limb motor	Lower limb sensory	Total
Axonal	56	19	138	66	279
Demyelinating	7	18	12	2	39
Mixed	4	6	7	5	22

Type of neuropathy

Among patients with PN (n=106), sensorimotor neuropathy was the most prevalent type, affecting 70.7% (n=75) of individuals. This was followed by pure motor neuropathy in 22.6% (n=24) and pure sensory neuropathy in 6.6% (n=7). The majority of sensorimotor cases exhibited an axonal pattern, while a significant portion also showed a mixed pattern. Pure motor neuropathy was predominantly axonal, with a smaller number displaying demyelinating or mixed features. Pure sensory neuropathy was rare and primarily axonal in nature.

Michigan Neuropathy Screening Instrument (MNSI)

The MNSI history component identified 14 patients (12.06%) with ≥7 abnormal responses, the threshold for a positive result. The most frequently reported symptom among these individuals was a burning sensation in both feet, noted by all patients with a positive history. However, the history component demonstrated limited diagnostic utility, identifying only 13.2% (n=14) of the 106 patients with electrophysiologically confirmed diabetic peripheral neuropathy (DPN).

In contrast, the MNSI examination component, considered positive when ≥3 abnormalities were detected, had a higher positivity rate of 51.7% (n=60), detecting 56.6% (n=60) of those with confirmed DPN. The most common clinical sign was the absence of the ankle jerk reflex, observed in 58 of the 60 patients with a positive examination. Importantly, all 14 patients who tested positive on both the history and examination components were confirmed to have DPN via nerve conduction studies.

Despite the higher sensitivity of the examination component compared to the history component, nearly 50% (n=53) of confirmed DPN cases remained undetected by the MNSI alone.

Comparison of the prevalence of neuropathy between diabetic and non-diabetic patients

Peripheral neuropathy was observed in 100% of diabetic patients (n=35), whereas it was present in 87.65% of non-diabetic patients (n=71 out of 81). Notably, all patients without peripheral neuropathy (n=10) were non-diabetic.

Regarding the pattern of nerve involvement, 68.57% (n=24) of diabetic patients exhibited neuropathy involving both upper and lower limbs, compared to 51.85% (n=42) of non-diabetic patients. In contrast, lower limb-only involvement was observed in 31.43% (n=11) of diabetics and 35.8% (n=29) of non-diabetics. The association between diabetes and the extent of limb involvement was statistically significant (p<0.001), indicating a higher burden of widespread neuropathy among diabetic individuals with CKD5D.

Association of peripheral neuropathy with sociodemographic and clinical parameters

Peripheral neuropathy was significantly more prevalent among diabetic patients (100%, n=35) compared to non-diabetics (87.6%, n=71 out of 81), with a statistically significant association (p=0.030). A sex-based difference was also observed, with a higher prevalence in males (95%, n=76 out of 80) than in females (83.3%, n=30 out of 36) (p=0.038).

However, no statistically significant association was found between peripheral neuropathy and age group (p=0.405), with PN observed in 100% (n=2) of those aged <20 years, 88.4% (n=38 out of 43) of those aged 20-40 years, 89.8% (n=44 out of 49) of those aged 40-60 years, and 100% (n=22) of those over 60 years. Similarly, dietary pattern (non-vegetarians: 91.0%, n=101 out of 111; vegetarians: 100%, n=5) (p=0.483) and the use of ESA (users: 93.1%, n=54 out of 58; non-users: 89.7%, n=52 out of 58) (p=0.508) showed no significant associations with PN.

The duration of hemodialysis also did not show a significant correlation with peripheral neuropathy (p=0.858). Among those on hemodialysis for <1 year, 91.8% (n=78 out of 85) had PN; for one to three years, 88.2% (n=15 out of 17); for three to five years, 100% (n=6); and for >5 years, 87.5% (n=7 out of 8). Regarding hemodialysis frequency, all patients receiving once-weekly hemodialysis (n=3) had PN; 92.3% (n=84 out of 91) of those receiving twice-weekly hemodialysis had PN; and 86.4% (n=19 out of 22) of those on thrice-weekly hemodialysis had PN. However, the frequency of hemodialysis sessions per week was not significantly associated with PN (p=0.581).

Importantly, lower serum albumin levels were significantly associated with the presence of peripheral neuropathy (p=0.017). Among those with serum albumin <30 g/L, 75% (n=12 out of 16) had PN; in the 30-40 g/L group, 96.1% (n=74 out of 77); and in the >40 g/L group, 87.0% (n=20 out of 23) had PN. This suggests a potential link between poor nutritional status and increased neuropathy risk in patients with CKD5D. The association of peripheral neuropathy with sociodemographic, clinical, and laboratory parameters is summarized in Table 5.

**Table 5:** Association of peripheral neuropathy with sociodemographic, clinical, and laboratory parameters PN: peripheral neuropathy; ESA: erythropoiesis-stimulating agent; HD: hemodialysis; iPTH: intact parathyroid hormone; NA: not applicable

Variable	Category	PN, yes (n)	PN, no (n)	P-value	Test statistic (χ² or Fisher's)
Diabetes	Yes	35	0	0.03	Fisher’s exact p=0.030
	No	71	10	NA	NA
Age group (years)	<20	2	0	0.405	χ²=2.91, df=3
	20–40	38	5	NA	NA
	40–60	44	5	NA	NA
	>60	22	0	NA	NA
Sex	Male	76	4	0.038	χ²=4.28, df=1
	Female	30	6	NA	NA
Diet	Non-vegetarian	101	10	0.483	Fisher’s exact p=0.483
	Vegetarian	5	0	NA	NA
ESA use	Yes	54	4	0.508	χ²=0.44, df=1
	No	52	6	NA	NA
Duration on HD (years)	<1	78	7	0.858	χ²=0.57, df=3
	1–3	15	2	NA	NA
	3–5	6	0	NA	NA
	>5	7	1	NA	NA
Frequency of HD/week	Once	3	0	0.581	χ²=1.08, df=2
	Twice	84	7	NA	NA
	Thrice	19	3	NA	NA
Hemoglobin (g/dL)	7–9	47	2	0.517	χ²=1.34, df=2
	9–11	30	4	NA	NA
	Less than 7	23	3		NA
Potassium (mEq/L)	<3.5	6	5	0.764	χ²=0.52, df=2
	3.5–5.5	65	1	NA	NA
	>5.5	36	4	NA	NA
Phosphorus (mg/dL)	<4.5	35	3	0.84	χ²=0.36, df=2
	4.5–7.5	54	3	NA	NA
	>7.5	17	0	NA	NA
iPTH (pg/mL)	<50	5	0	0.362	χ²=2.02, df=2
	50–450	73	9	NA	NA
	>450	28	1	NA	NA
Serum albumin (g/L)	<30	12	4	0.017	χ²=8.12, df=2
	30–40	74	3	NA	NA
	>40	6	0	NA	NA
Vitamin B12 (pg/mL)	<200	20	5	0.256	χ²=4.62, df=3
	200–500	21	1	NA	NA
	500–800	40	0	NA	NA
	>800	16	0	NA	NA
HbA1c (%)	6.5–7.5	9	0	0.345	χ²=2.13, df=2
	7.5–8.5	13	0	NA	NA
	>8.5	9	0	NA	NA

## Discussion

This study presents the first comprehensive evaluation of the prevalence and clinical characteristics of PN among patients undergoing maintenance hemodialysis in Nepal. The findings contribute important insights into the epidemiological, clinical, and electrophysiological profiles of this vulnerable population, highlighting key areas for clinical focus and future research. The mean age of the study participants was 44.16 years (SD=15.47 years), which is considerably lower than the average age of 55 years reported in a similar study conducted in Switzerland [14]. This notable age difference may be attributed to a combination of sociocultural, environmental, and healthcare-related factors. In developing countries such as Nepal, limited access to preventive care, delayed diagnosis of CKD, and socioeconomic pressures may lead to earlier disease onset and progression compared to developed nations.

A male predominance was observed in the study cohort, with approximately two males for every female. This is consistent with findings from an Indian study examining sociodemographic and risk factor profiles in patients with end-stage renal disease [15]. The gender disparity may be influenced by cultural and socioeconomic norms in Nepal, where males often have greater access to healthcare services and decision-making authority regarding medical treatment. Additionally, this imbalance may reflect gender differences in health-seeking behavior and occupational exposure to CKD risk factors. The majority of participants were within the economically productive age group of 20-60 years. However, only 31% reported engagement in steady employment, indicating a significant socioeconomic burden posed by CKD in this population. This finding highlights the need for comprehensive rehabilitation and social support programs for patients receiving renal replacement therapy.

Regarding the etiology of CKD, the most common category was "undetermined," followed by diabetic nephropathy, which accounted for 27.6% of cases. This contrasts with global patterns, where diabetes mellitus is frequently identified as the leading cause of CKD [16]. The discrepancy may result from underdiagnosis of diabetes or glomerular diseases, and the limited availability of renal biopsies and diagnostic tools in low-resource settings. Similar concerns have been raised in other underserved populations, including those in Africa, where delayed presentation and limited diagnostic infrastructure hinder accurate etiological classification of kidney disease [17]. Strengthening diagnostic capabilities in Nepal could facilitate earlier intervention and improve disease characterization.

PN was found to be highly prevalent in our cohort, affecting 91.4% of patients. This is consistent with prior reports from Turkey and India, which showed neuropathy prevalence rates of 97% and 87.5%, respectively, among dialysis patients [18,19]. Our findings reinforce the view that PN remains a common and underrecognized complication of CKD, even in patients receiving regular hemodialysis. Notably, we did not observe a significant association between the frequency of dialysis sessions and the presence of neuropathy. This suggests that mechanisms beyond dialysis clearance, such as persistent accumulation of uremic toxins, ongoing microvascular damage, or chronic systemic inflammation, may play a more critical role in the development and persistence of uremic neuropathy.

All diabetic patients undergoing dialysis exhibited PN, while 87.7% of non-diabetic patients were also affected. This finding supports the well-established synergistic role of diabetes and uremia in promoting nerve injury [20,21]. The most commonly reported neuropathic symptom was a burning sensation in the feet, which was universally present among patients who screened positive. Clinically, the most frequent finding was absent ankle jerk reflex, observed in 67.2% of participants. This is consistent with the literature, which identifies the Achilles tendon reflex as a sensitive early indicator of distal large fiber neuropathy in CKD [21-23].

Our results emphasize the importance of comprehensive neurological evaluation in dialysis patients. The Michigan Neuropathy Screening Instrument identified only 12.1% of cases, while clinical examination revealed 56.6%. In contrast, nerve conduction studies detected a significantly greater number of affected individuals, confirming their role as the gold standard for diagnosis [22,24]. The substantial diagnostic yield of nerve conduction studies underscores the need for their routine use, particularly in centers where clinical assessment alone may underestimate disease prevalence.

Electrophysiological evaluation revealed that axonal neuropathy was the predominant form. In our study population, 62.3% of patients exhibited neuropathy involving both upper and lower limbs, while 37.7% had involvement limited to the lower limbs. No cases were observed with exclusive upper-limb involvement, which is consistent with the classical length-dependent pattern characteristic of uremic neuropathy [25]. These findings support the utility of lower-limb nerve conduction studies as an effective screening tool, particularly in resource-limited environments where full-limb studies may not be feasible.

A comparison of diabetic and non-diabetic subgroups revealed that 68.6% of diabetic patients had both upper and lower limb involvement, compared to 51.9% of non-diabetics, with a statistically significant difference (p<0.001). The common peroneal nerve was the most frequently affected motor nerve, while the sural nerve was the most commonly involved sensory nerve. This distribution pattern is in agreement with previously published studies on peripheral neuropathy in CKD [20]. Sensorimotor neuropathy was the most prevalent subtype, affecting 71% of patients, followed by pure motor neuropathy in 24% and pure sensory neuropathy in 6%. These findings are typical of the distal axonal sensorimotor polyneuropathy frequently associated with advanced kidney disease [20,25].

The significant association between diabetes and peripheral neuropathy in dialysis patients observed in our study is consistent with prior research, which has demonstrated the cumulative impact of these two conditions on nerve health [21]. Although some studies have reported a correlation between elevated serum potassium levels and progression of neuropathy [26], our study did not observe such an association. A randomized controlled trial evaluating potassium restriction in CKD patients showed potential improvements in nerve function [27], but this effect may not have been replicated in our cohort due to differences in diet, compliance, or study design.

Erythropoiesis-stimulating agent therapy did not show a statistically significant relationship with neuropathy in our study population. This is in agreement with findings from other clinical trials, where such agents improved anemia-related outcomes but did not translate into measurable improvements in neuropathic symptoms or quality of life [28]. The results suggest that factors such as chronic toxin exposure, vascular compromise, and systemic inflammation may play more central roles in the pathogenesis of neuropathy in CKD.

An important and novel observation in our study was the statistically significant association between low serum albumin levels and the presence of peripheral neuropathy (p=0.017). Previous studies have shown that hypoalbuminemia, a marker of malnutrition and systemic inflammation, is associated with increased severity of diabetic complications, including neuropathy and retinopathy [29]. Additionally, lower albumin levels have been correlated with impaired nerve conduction velocities in patients with type 2 diabetes mellitus [29]. These findings suggest that nutritional status may directly influence nerve health and that addressing protein-energy malnutrition could have therapeutic implications in dialysis-associated neuropathy.

Although most patients in our cohort had serum vitamin B12 levels within the normal reference range, we acknowledge that this does not entirely exclude the possibility of a functional deficiency. Biomarkers such as methylmalonic acid and homocysteine are more sensitive indicators of tissue-level B12 deficiency and may be useful in future research to further investigate the role of vitamin B12 in this patient population [30].

Limitations

This study has several limitations that should be considered when interpreting its results. First, the hospital-based cross-sectional design may limit the generalizability of findings to the broader dialysis population in Nepal. Second, in five patients, lower-limb nerve conduction studies could not be performed due to the presence of significant edema, which may reduce the accuracy of the overall neuropathy prevalence estimate. Third, because baseline neurological assessments were not performed before the initiation of hemodialysis, it is difficult to determine whether neuropathy developed before or after dialysis initiation. Despite these limitations, the study underscores the importance of early screening and interdisciplinary management of neuropathy in patients with end-stage renal disease.

Future directions

Future research should focus on large-scale, multicenter, and prospective studies to validate the findings of this study and better understand the natural history and progression of neuropathy in dialysis patients. Investigations into the effectiveness of nutritional support, toxin clearance strategies, and neuroprotective interventions will be essential in developing evidence-based management protocols. Establishing standardized clinical guidelines that integrate neurological assessment into routine nephrology care could improve long-term outcomes and quality of life for patients with CKD undergoing dialysis.

## Conclusions

PN is highly prevalent among hemodialysis patients in Nepal, affecting over 90% of this population. Nutritional status, as indicated by low serum albumin levels, plays a significant role in neuropathy development and represents an important target for intervention. Given the limited sensitivity of clinical screening tools, routine use of nerve conduction studies is essential for early and accurate diagnosis. A multidisciplinary approach that includes nutritional support, regular screening, and collaboration between nephrologists, neurologists, and rehabilitation specialists is crucial to improving the quality of life and clinical outcomes for patients with CKD undergoing dialysis.
